# Advances in Wood and Wood Polymer Composites

**DOI:** 10.3390/polym18111403

**Published:** 2026-06-05

**Authors:** Antonios N. Papadopoulos

**Affiliations:** Department of Natural Environment and Climate Resilience, Democritus University of Thrace, 66100 Drama, Greece; antpap@neclir.duth.gr

## 1. Introduction

Wood composites are synthetic materials that are assembled by combining lignocellulosic raw materials like wood fibers, particles, veneers, strands, or agricultural residues with proper binders and additives under controlled manufacturing conditions. They have seemingly become even more important in modern construction and manufacturing because they facilitate the use of virgin and recycled biomass. Unlike solid wood, which can contain natural defects and exhibit a great deal of natural variability in performance, wood composites can be designed to have more consistent and predictable properties, so they tend to be very dependable for both structural and non-structural applications.

One of the greatest advantages of wood composites is that they are incredibly versatile in terms of their design and production. By choosing the right kind of raw material, binder system, manufacturing process, and even panel setup, manufacturers can tune these materials so that they fit particular end-use needs and meet performance targets people truly care about. In other words, aspects like strength, rigidity, dimensional stability, resistance to moisture, thermal insulation, and long-term durability can all be adjusted depending on the ultimate application. Because of this kind of flexibility, wood composites can be used in numerous sectors—furniture manufacturing, interior decoration, flooring, packaging, automotive parts, structural building applications, etc.—while still staying within the required mechanical or structural performance limits.

Wood composites also play a crucial role in the sustainable and efficient use of forest resources. They can be created using low-quality timber, wood residues, sawmill byproducts, and even agricultural lignocellulosic waste materials that might otherwise remain unused or be discarded. So, in practice, making wood composites helps reduce the need for solid wood products and foster the more effective use of what is already available. This capacity aligns with sustainable forest management, and it can also help reduce environmental impacts linked to over-harvesting of natural timber. Thus, wood composites appear to be an environmentally and economically appealing alternative to conventional solid wood materials for numerous modern applications.

This Special Issue, Advances in Wood and Wood Composites, is a continuation of the three previous successful series, namely, ‘Advances in Wood Composites I, II and III’ [1,2,3] and presents recent progress and development in regard to refining and enhancing the overall performance of wood composites. This Special Issue, which collects 10 original, high-quality research papers, provides selected examples of recent advances in wood and wood composites.

## 2. An Overview of the Published Articles

An interesting review on wood/dynamic covalent polymer network composites was published in this Special Issue [4]. This review summarizes recent advances in wood/DCPN (dynamic covalent polymer network) composites and discusses two main fabrication routes: impregnating delignified wood with DCPN and blending DCPNs with wood powder. Overall, wood/DCPN composites combine the characteristics of wood and these dynamic DCPNs, and they might become a more efficient, eco-friendly, and sustainable way of processing and utilizing wood. In the future, researchers might develop new DCPNs with specialized functionalities while also advancing wood-processing and modification techniques and uncovering new ways of blending wood with dynamic covalent polymers in a more seamless manner. These pathways are likely to offer real opportunities for further progress in this area. More directly, converting the molecular network of wood into DCPNs in situ by introducing reversible chemical bonds appears to be a cleaner, less roundabout way of preparing wood/DCPN composites, but it remains challenging in practice.

Wilczyński et al. [5,6] studied the rheology of wood plastic composite extrusion. Rheological data are basically the backbone for good design in polymer-processing tasks such as extrusion. Rheological property tests are expensive and very time consuming. Moreover, they are often performed under conditions (temperature, pressure, and shear rate) that are drastically different from those in processing. Accordingly, inexpensive and fast methods of acquiring rheological data are required, along with methods that can be applied under processing conditions in a more practical way.

In the first part of the study [5], research on the rheology of WPCs was carried out under lab and production conditions. On the lab side, the experiments were performed using high-pressure capillary rheometry (HPCR), and they relied on the Melt Flow Index (MFI). For the production conditions, i.e., the online tests, the setup was different because extrusion die pressure and extrusion throughput were measured instead. Then, the MFI-based viscosity and online viscosity were evaluated against the background of the HPCR’s viscosity to compare them. It was concluded that the MFI method of determining viscosity and the online tests may be rather reasonable alternatives to HPCR measurements, although there is still some uncertainty in practice. The authors also sought to evaluate this concept based on theoretical and experimental studies of the extrusion process and see what came of it.

In the second part of the study, viscosity data and models were used for extrusion process simulations [6]. Experimental studies of the process were performed, and the results were used to evaluate the models applied. The authors concluded that the idea of using only limited MFI measurements—and the online kind, too—can be applied in engineering to quickly evaluate the rheological behaviors of WPCs. In cases where available databases do not offer this information, this approach can be considered workable and practical.

Yunlu [7] examined the effect of using goat horn powder (GHP) as reinforcement on the hardness, microstructure, and mechanical properties of wood-like polyurethane composites. In this work, GHP, a keratin-based animal waste product, was inserted into a polyurethane matrix using weight fractions of 5, 10, 15, 20, and 25 wt.%. The authors showed that GHP can serve as a functional, sustainable strengthening element and increase toughness and hardness while also aiding in the handling of environmental waste.

Nedic et al. [8] provided compound-resolved evidence from a full-scale industrial MDF dryer. Their results showed that the cleaned stack signature is not spread out but instead concentrated in just a few chemically meaningful marker compounds. α-Pinene, 3-carene, limonene, methanol, and formaldehyde made up more than 80% of the resolved VOC mix, demonstrating that even a disorganized industrial emission profile can still be read through a limited chemotype.

In the wood-processing industry, wood adhesives are critical, as they greatly aid bonding. Still, the older formaldehyde-based adhesives pose health risks and depend on non-renewable resources. Liu et al. [9] aimed to develop a bio-based wood adhesive with excellent water resistance, focusing on environmentally friendly solutions. They reported that oxidation of starch is a fairly useful approach to extracting biomass aldehyde compounds, which can then be used to react with lignin, consequently yielding environmentally friendly biomass adhesives. But the oxidation step must be completed carefully and with considerable control so that the level of over-oxidation remains minimal. Such over-oxidation is often tied to the appearance of carboxyl groups as opposed to the more active aldehyde groups. In other words, it can shift things away from where you actually want them, so how you carry out the step is critical.

Dudeva et al. [10] investigated and evaluated the performance of ammonium bisulfite and urea–metabisulfite as formaldehyde scavengers for a low-molar-ratio UF resin (F/U = 1.06) at 1, 3, and 5 wt% (based on dry UF resin solids) used for MDF-panel manufacturing. The results showed that sulfite-based scavengers can be incorporated into low-molar-ratio UF adhesives in order to obtain ultra-low-formaldehyde MDF while still keeping the main panel properties nearly intact.

Stanciu et al. [11] presented new challenges in assessing the acoustic properties of coating polymers. The novelty of their research mostly lies in the fact that it evaluates the acoustic and dynamic parameters of resonant spruce-wood boards. These boards are treated with varnishes, each with different chemical properties (e.g., oil-based, spirit, and nitrocellulose varnish). The authors concluded that the acoustic performance of a soundboard can be tailored by selecting the appropriate varnishing system and number of layers to apply.

Deknes et al. [12] studied the efficiency of near-infrared spectroscopy in quantifying lignin content in black-liquor-impregnated reforestation wood. They employed near-infrared (NIR) spectroscopy coupled with partial least-squares regression (PLSR) to predict cellulose and lignin content in sapwood and heartwood of *Eucalyptus urophylla* and sapwood of *Pinus taeda*, all impregnated with black liquor under high pressure. Their findings confirm the efficacy of NIR-PLSR as a nondestructive, reliable alternative to conventional chemical analyses, with implications for improving quality control and decision-making in the wood treatment industry.

Finally, Tian et al. [13] focused on theoretically deriving a moisture content distribution model for cylindrical wood components, which was subsequently validated through experiments. The model comprehensively considers the temporal evolution of and spatial variation in moisture content.

## 3. Conclusions

It can be stated, perhaps with confidence, that the field of wood and wood composites is dynamic but also very promising, and the future looks exceptionally bright. This text provides a short glimpse of the kind of changes and novelty expected to influence the field soon. Research labs across the world are still carrying out pioneering work, and yet new difficulties, methods, and ideas keep arising, emphasizing how fast wood composite research is moving and how truly engaging it still feels.

After the success of the earlier Special Issue about “Advances in Wood and Wood Composites,” a second is now available and open for submissions. The aim of this fresh edition is to keep shining a light on recent scientific progress and inventive technologies, along with emerging trends that are still advancing the field, even with respect to expanding its potential applications.
